# Assessment of radiation hazards from natural radionuclide activity in quarry sites and surrounding soils in Osun State, Southwest Nigeria

**DOI:** 10.1038/s41598-025-02239-w

**Published:** 2025-06-02

**Authors:** Olubusayo F. Oladejo, Stephen F. Olukotun, Abdulfatai B. Rufai, Manny Mathuthu

**Affiliations:** 1 Department of Physics, Osun State University, Osogbo, Nigeria; 2 Department of Physics and Engineering Physics, Obafemi Awolowo University, Ile-Ife, Nigeria; 3 Center for Applied Radiation Science and Technology (CARST), North-West University, Mahikeng Campus, Mahikeng, South Africa; 4 Department of Plant Biology, Osun State University, Osogbo, Nigeria

**Keywords:** Dose rates, Gamma-ray spectroscopy, NORMs, Quarry products, Radiation risk indices, Radium equivalent, Environmental sciences, Natural hazards, Health occupations, Risk factors

## Abstract

This study assessed the radioactivity levels of naturally occurring radioactive materials (NORMs), specifically ^40^K, ^232^Th, and ^238^U, in quarry products and surrounding soils from four major commercial quarry sites in Osun State, Nigeria, using gamma spectroscopic technique. The average activity con^40^centrations of K, ^232^Th, and ^238^U in the quarry products are 332.55 ± 28.8, 9.72 ± 1.0, and 11.35 ± 1.0 Bq/kg, respectively, all below global averages. Radiation risk indices, including radium equivalent levels, effective dose rates, absorbed dose rates, gamma radioactivity level index, internal and external hazard indices, and gonadal dose equivalent, were evaluated. The radium equivalent levels ranged from 48.77 to 53.25 Bq/kg, well below the global limit of 370 Bq/kg, while the absorbed dose rates were 40% lower than the global average. Total effective dose rates and hazard indices were also significantly below safety thresholds, indicating negligible radiation threats. Statistical analysis revealed strong correlations between ^40^K and radiological indices, showing its dominant influence on overall radiation risk. Correlation and hierarchical cluster analysis further affirmed that the evaluated radiological risks were dependent on the radionuclides measured concentrations. The results conclude that both the quarry products and surrounding soils pose no significant radiation hazards, rendering them safe for construction and human activities.

## Introduction

The process of extracting natural rocks and minerals from the continental crust to obtain various materials, including stone, gravel, sand, and other construction materials, is known as quarrying. Quarrying is typically carried out in open-pit mines, excavations, or quarries^1,2^. It involves heavy machinery, explosives, and other tools to break, extract, and process the materials. The products obtained through this process are used for constructing roads, bridges, buildings, and infrastructure, as well as for landscaping and architectural purposes. Quarry products are also used for decoration, porcelain fixtures, and ornamental and monumental materials due to their aesthetic appeal and easy maintenance. However, the health and environmental effects of quarrying activities should not be underestimated^3–7^.

Quarry products, depending on their geological formation and geographical conditions, contain varying levels of natural radionuclides such as thorium, potassium, and uranium, which are generally spread throughout the Earth’s crust. The distribution of these radionuclides is different in different quarry ecosystems, and their activity concentrations are connected with the geology of each lithologically divided area^8–12^. Quarry products have been identified as probable hosts for elevated elemental concentrations of thorium and uranium, where they are typically available in concentrations of 1–10 ppm and 3–30 ppm, respectively, in the Earth’s crust^13^. These increased concentrations can be traced to the differentiation and crystallization of magma, leading to thorium and uranium being concentrated in silica-rich products^14^. A higher radiation level in quarry products has also been established^15,16^.

The extraction and processing of quarry products can cause contamination of surface soils and nearby aquatic environments by natural radionuclides^17^. Contaminated soils expose humans and other living organisms to high levels of radiation through gamma radiation absorption or inhalation of carcinogenic radon gas and its progeny^18^. Prolonged exposure to elevated gamma radiation levels can result in acute diseases, including kidney, lung, and digestive system cancers^19–21^. Investigating the radionuclide content of quarry products is essential to assess and prevent potential radiation exposure. Periodic assessments of environmental radiation levels in quarry products can help determine activity levels and assess any potential radiological hazards to workers and residents in and around quarry areas, as well as to individuals living in houses or rooms constructed or decorated with quarry products. This is crucial for controlling radiation threats and achieving sustainable development goals.

Due to their abundant natural resources and favorable geological conditions, some states in Nigeria have thriving quarry industries, which play a vital role in the country’s infrastructure development, including road construction, building projects, and urban development. Nigerian quarries produce materials essential to the construction industry, such as granite, limestone, sand, gravel, quarry dust, and crushed stones. These products are used in the construction of various structures—residential, commercial, industrial, religious, recreational, and educational buildings, among others—highlighting the importance of periodically measuring their activity concentrations and assessing associated radiation hazards to ensure public safety.

Evaluating the activity concentrations and radiation risks in quarries across Nigeria has drawn significant attention from researchers aiming to assure the safety of quarry workers, nearby communities, and the environment^9–11,17,22–27^. Despite this interest, insufficient radiological assessments of quarry products from specific sites have been reported. This research aims to examine the activity levels of NORMs in selected commercial quarry sites (the major distributors of quarry products) in Osun State, Southwestern Nigeria, and its environments and to evaluate the potential radiological risks they may present. Additionally, the study will assess the possible influence of quarrying activities on surrounding soils.

## Materials and methods

### Research area

**Fig. 1:**
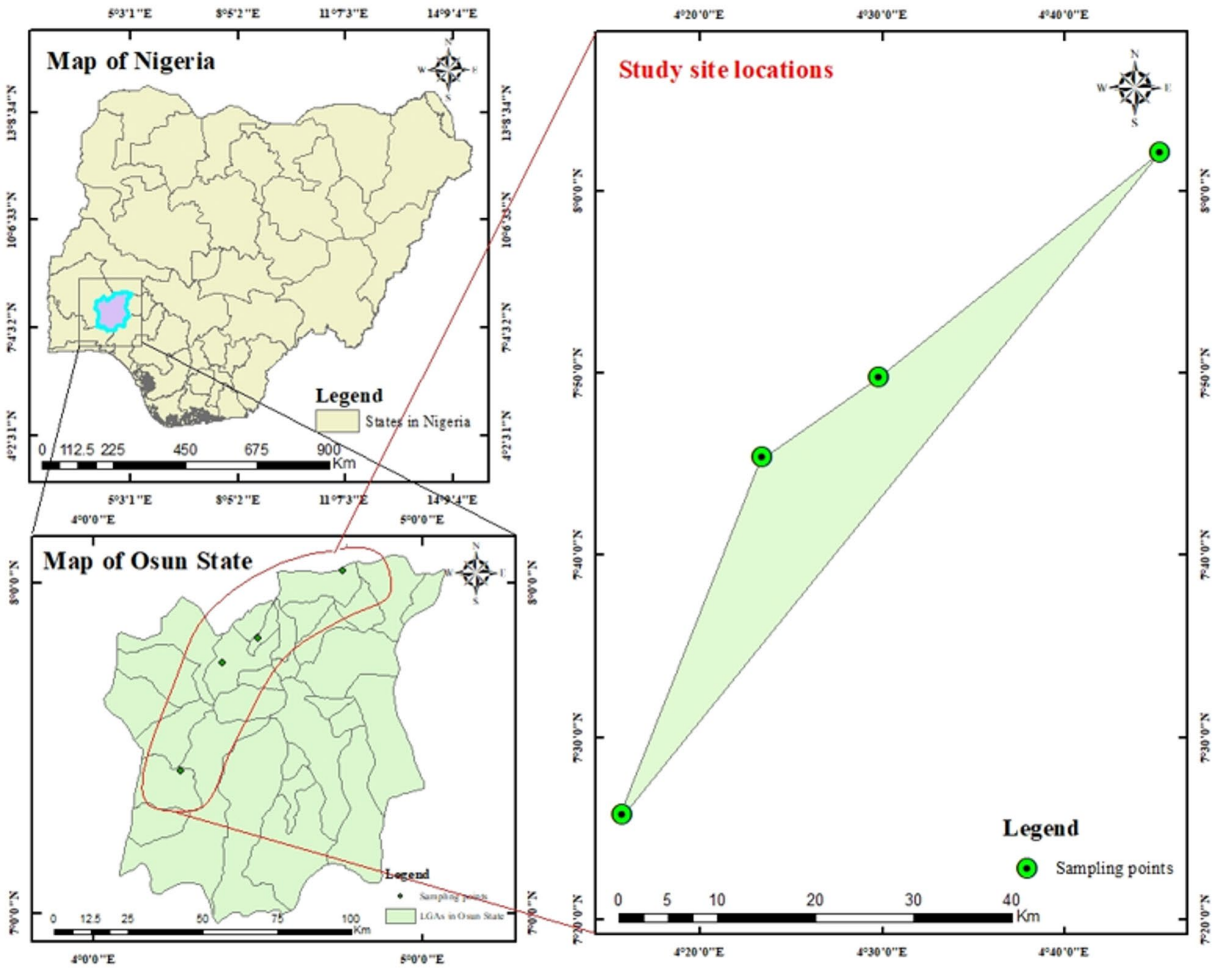
Locations of the studied site on the map.

Osun State is one of the six states in the southwestern part of Nigeria. It spreads over an area of approximately fourteen thousand, eight hundred square kilometers and lies within latitudes 6.984716 and 8.081845 and longitudes 4.049945 and 4.895892^28^. The climate of Osun State is typified by a lengthy rainy season between March and July, a dry season between July and August, a short wet season between August and November, and a harmattan season from November to March. Geologically, the study area is underlain by Precambrian rocks of the basement complex of Nigeria. The deep weathering profiles of Osun State, which consist of various topographic features caused by the differential rock degradation and the accumulation of debris acted upon by exogenic geomorphic forces, have resulted in significant mineral deposits such as laterites, feldspars, talc, columbite, cassiterite, iron ore, granite, tourmaline, and mica^29–31^. This geological diversity has not only shaped the landscape of Osun State but also plays a crucial role in resource exploration and economic activities. The presence of these mineral deposits, especially granites has attracted quarrying activities in various parts of the state, the product of which is widely used in construction for residential and commercial purposes across the state and beyond. The use of these quarry products is a course for concerns about potential radiological hazards it might pose to the public, making their assessment crucial to this study. The map and coordinates of the study areas are provided in Fig. 1; and Table 1, respectively.

### Sample collection and preparation

Forty samples (five quarry products obtained randomly from each quarry site and five soil samples from areas within 2–5 km away from the quarry sites) were collected from four quarry sites and their surroundings. Each sample was kept in a labeled polyethylene bag and sealed. All samples were transported to the Pollution Research Laboratory, Department of Physics, Osun State University, Osogbo, for further processing. Wet soil samples were dried to constant weight at ambient temperature in the laboratory, and rock samples were crushed with a hammer on a metal base. The hammer and the metal base were thoroughly cleaned and properly dried before use with fresh samples to prevent cross-contamination. Crushed samples were sieved using a 4-millimeter mesh, and subsequently, 100 g of each sample was weighed using an OHAUS Adventure Pro AV264 digital balance. The samples were placed into polyvinyl chloride (PVC) containers, sealed airtight, and stored for four weeks to allow short-lived daughter radionuclides and long-lived parent radionuclides to reach secular equilibrium. The activity levels of NORMs in the samples were measured using a calibrated gamma-ray spectrometer at the Radiation Laboratory, Ladoke Akintola University of Technology, Ogbomoso, Nigeria.

**Table 1:** Geographical locations of the study area.

S/*N*	Sample tag	Coordinates	
		Latitude	Longitude
1	QSA	8.03585	4.754210
2	QSB	7.82980	4.496376
3	QSC	7.756881	4.390136
4	QSD	7.429095	4.261811

### Gamma spectrometry

The gamma spectrometer at LAUTECH is equipped with a 3” by 3” sodium iodide, thallium-activated (NaI[Tl]) scintillation detector, manufactured by Princeton Gamma Technology, United States of America (USA), and housed in a lead shield to prevent background radiation. Each assay was put on the detector for 600 min (10 h) to determine the activity levels of ^232^Th, ^238^U, and ^40^K in Bq/kg using the count spectra. Analytical Quality Control Services (AQCS) USA standards, containing 10 radionuclides with gamma emitters of energies ranging from 59.54 to 1836 keV, were used to calibrate the energy resolution and efficiency of the detector. The peak of ^234^Th (63.3 keV) was used to determine the activity level of ^238^U, while the average activity levels of ^214^Pb (295.3 keV) and ^214^Bi (1764 keV) were used to determine the radioactivity levels of ^226^Ra. The activity concentration of ^232^Th was determined from the average concentrations of ^228^Ac (911.1 keV), ^208^Tl (2614.7 keV), and ^212^Pb (238.6 keV). The energy of 1460.0 keV was used to identify ^40^K. The radioactivity level of ^235^U was ascertained from the 185.7 keV gamma line, which was corrected by subtracting the contribution from the 186.2 keV of ^226^Ra using Eq. (1).

1 $$\:A{(}^{235}U)=\frac{{N}_{186}-A{(}^{226}Ra).{f}_{E}{(}^{226}Ra).{n}_{186}.M.{T}_{c}}{{n}_{186}.{f}_{E}{(}^{235}U).M.{T}_{c}}$$

Where $\:{N}_{186}$ is the cumulative count for the 186 keV doublets. $\:{n}_{186}$ is the detection efficiency of the 186 keV line and $\:A{(}^{235}U)$ and $\:A{(}^{226}Ra)$ are the activity of ^235^U and ^226^Ra, respectively. $\:{{f}_{E}{(}^{226}Ra)\:\:\text{a}\text{n}\text{d}\:f}_{E}{(}^{235}U)$ are probabilities of the emission of the gamma lines of ^226^Ra and ^235^U, respectively. The mass of the sample is M and $\:{T}_{c}$ is the counting time.

2 $$\:LDA=\frac{1.645\sqrt{{N}_{B}}}{{f}_{E}.n\left(E\right).{T}_{c}.M}$$

Equation (2) was used to evaluate the least detectable activity (LDA) for each NORM ^226^Ra, ^232^Th, and ^40^K. Where 1.645 is the statistical coverage factor at a 95% confidence level and $\:{N}_{B}$ is the background counts at the region of interest. The probability of gamma emission is $\:{f}_{E}$, the efficiency of the photopeak is $\:n\left(E\right)$ and M is the sample mass. The LDA for each of the radionuclides was calculated as 0.12 Bq kg^−1^ for ^226^Ra, 0.11 Bq kg^−1^ for ^232^Th, and 0.9 Bq kg^−1^ for ^40^K^11,32^.

### Evaluation of radiation risk of quarry products and the surrounding soils

An extensively used radioactive parameter to assess radiation health risks is the radium equivalent level (Ra_eq_). Ra_eq_ was employed to evaluate the exposure to gamma radiation from the sampled products^11,20^ It is calculated using the details provided in serial number 1 of Table 2, where C_Th_, C_U_, and C_K_, and represent the activity levels of ^232^Th, ^238^U, and ^40^K, respectively.

The damage caused by radiation to tissues and organs is a function of the absorbed dose rate, which refers to the imparted energy per unit mass of irradiated materials. The absorbed dose rate (D_R_), attributable to the emission of gamma rays from the radionuclides present in the studied quarry products, was calculated using figures and equations from the US Environmental Protection Agency^33^, European Commission^34^, and UNSCEAR^12^. For a consistent distribution of NORMs, dose conversion factors of 0.92, 1.1, and 0.080 nGy/h per Bq/kg for ^238^U, ^232^Th, and ^40^K, respectively, were used to convert radioactivity levels to the rate of absorbed dose in the air at 1 m above ground level^20^. Serial number 2 of Table 2 provides the details used to calculate D_R_.

Associated with D_R_ are the indoor, outdoor, and total effective doses. The outdoor and indoor exposure to gamma radiation depends on geographical location, the area’s geological characteristics, and the construction materials used. The outdoor effective dose (A_doutdoor_) represents the amount of ionizing radiation a person might receive while spending time outdoors per year. A_doutdoor_ is calculated using a 0.2 occupancy factor and a 0.7 Sv/Gy conversion coefficient, which converts the absorbed dose in the air to the effective dose absorbed by adults. Similarly, the indoor effective dose (A_dindoor_) reflects the cumulative indoor ionizing radiation exposure per year, using a 0.8 occupancy factor. The total effective dose (A_dTotal_) is the sum of A_doutdoor_ and A_dindoor_^11,20^. The evaluations of A_doutdoor_, A_dindoor_, and A_dTotal_ were performed using the equations provided in serial numbers 3–5 of Table 2.

**Table 2:** Radiological parametric quantities, evaluating expressions, units, and recommended limits.

S/*N*	Parametric quantity	Evaluating expression	Unit	Recommended limit
1	$\:{Ra}_{eq}$	$\:{C}_{U}+1.43{C}_{Th}+0.077{C}_{K}$	$\:{Bq\:kg}^{-1}$	$\:<370$ [16]
2	$\:{D}_{R}$	$\:{0.92C}_{U}+1.1{C}_{Th}+0.080{C}_{K}$	$\:{nGy\:h}^{-1}$	$\:<84$ [16]
3	$\:{A}_{doutdoor}$	$\:{D}_{R}\times\:1227.24\:mSv(nGy{)}^{-1}h\:{y}^{-1}$	$\:mSv\:{y}^{-1}$	$\:<0.07\:$[16]
4	$\:{A}_{dindoor}$	$\:{D}_{R}\times\:4908.96\:mSv(nGy{)}^{-1}h\:{y}^{-1}$	$\:mSv\:{y}^{-1}$	$\:<0.41$ [16]
5	$\:{A}_{dTotal}$	$\:{A}_{doutdoor}+{A}_{dindoor}$	$\:mSv\:{y}^{-1}$	$\:<0.49$ [16]
6	$\:{H}_{\varvec{i}\varvec{n}}$	$\:\frac{{C}_{u}}{185\,Bq/kg}+\frac{{C}_{Th}}{259\,Bq/kg}+\frac{{C}_{K}}{4810\,Bq/kg}$		$\:<1$ [16]
7	$\:{H}_{\varvec{e}\varvec{x}}$	$\:\frac{{C}_{u}}{370\,Bq/kg}+\frac{{C}_{Th}}{259\,Bq/kg}+\frac{{C}_{K}}{4810\,Bq/kg}$		$\:<1$ [16]
8	$\:{I}_{\gamma\:}$	$\:\frac{{C}_{u}}{300\,Bq/kg}+\frac{{C}_{Th}}{200\,Bq/kg}+\frac{{C}_{K}}{3000\,Bq/kg}$		$\:<1$ ^16^
9	$\:I$	$\:\frac{{C}_{u}.{f}_{u}}{50\,Bq/kg}+\frac{{C}_{Th}.{f}_{Th}}{50\,Bq/kg}+\frac{{C}_{K}.{f}_{K}}{500\,Bq/kg}$		$\:<2$ ^16^
10	$\:{D}_{GE}$	$\:{3.09C}_{U}+4.18{C}_{Th}+0.314{C}_{K}$	$\:\mu\:Sv\:{y}^{-1}$	$\:<300$ ^16^
11	$\:ELC{R}_{outdoor}$	$\:{A}_{doutdoor}\times\:LE\times\:RF$		$\:0.29\:$ ^35^
12	$\:ELC{R}_{indoor}$	$\:{A}_{indoor}\times\:LE\times\:RF$		$\:1.16$ ^35^
13	$\:ELC{R}_{Total}$	$\:ELC{R}_{outdoor}+ELC{R}_{indoor}$		$\:1.45$ ^35^

For negligible radiation risk and the harmless use of the studied samples as building and construction materials, internal (H_in_) and external (H_ex_) radiation indices are evaluated. These radiological parameters are utilized to appraise the threat of gamma-emitted radiation to human health when carcinogenic radon and its short-lived progeny are inhaled, or when there is exposure to potential external radiation hazards^36–38^. To evaluate H_ex_, an assumption is made that radioactivity levels of 4810, 259, and 370 Bq/kg of ^40^K, ^232^Th, and ^238^U, respectively, contribute the same dose rate of gamma radiation. The expressions presented in serial numbers 6 and 7 of Table 2 are used to evaluate H_in_ and H_ex_.

The gamma radioactivity level index (I_γ_) is another index of gamma radiation risk, mainly used to determine the intensity of gamma-emitted radiation associated with specific radioactivity levels^23,34,39^. Details on the evaluation of the gamma radioactivity level index are provided in serial number 8 of Table 2.

Another factor influencing the absorbed dose indoors is the radioactivity levels of NORMs in building materials such as stones and granite, while outdoor-emitted radiation is effectively absorbed by walls. Accordingly, the indoor air dose rate will increase based on the activity levels of NORMs in the building materials. This factor is denoted by the activity utilization index (I), and it is evaluated as presented in serial number 9 of Table 2, where f_K_ (43.98%), f_Th_ (47.98%), and f_U_ (8.09%) are the fractional contributions of the total dose rate from ^40^K, ^232^Th, and ^238^U, respectively^36,39^.

Some key organs of concern in the body that can also be affected by gamma radiation include bone surface cells, gonads, and active bone marrow^16,40,41^. The gonadal dose equivalent per annum (D_GE_) for the exposed population to the studied quarry products was evaluated to assess the impact of gamma radiation doses from the activities of ^238^U, ^232^Th, and ^40^K on the gonads. D_GE_ is calculated using the expression in serial number 10 of Table 2.

Excess Lifetime Cancer Risk (ELCR) is used in radiation protection and environmental health to estimate the probability of an individual developing cancer over their lifetime due to exposure to carcinogenic agents, particularly ionizing radiation^35,42,43^. It is calculated using the expression in serial numbers 11, 12, and 13 of Table 2, where $\:LE$ is the life expectancy time and the life expectancy of a Nigerian use in this work is 55 years^43^ and $\:RF$ is the risk factor, that is the fatal cancer risk per Sievert. For low-dose background radiations which are considered to produce a stochastic effect, $\:0.05\:{Sv}^{-1}$ is used in this work for public exposure^44^.

## Results and discussion

### Levels of measured radioactivity

**Fig. 2:**
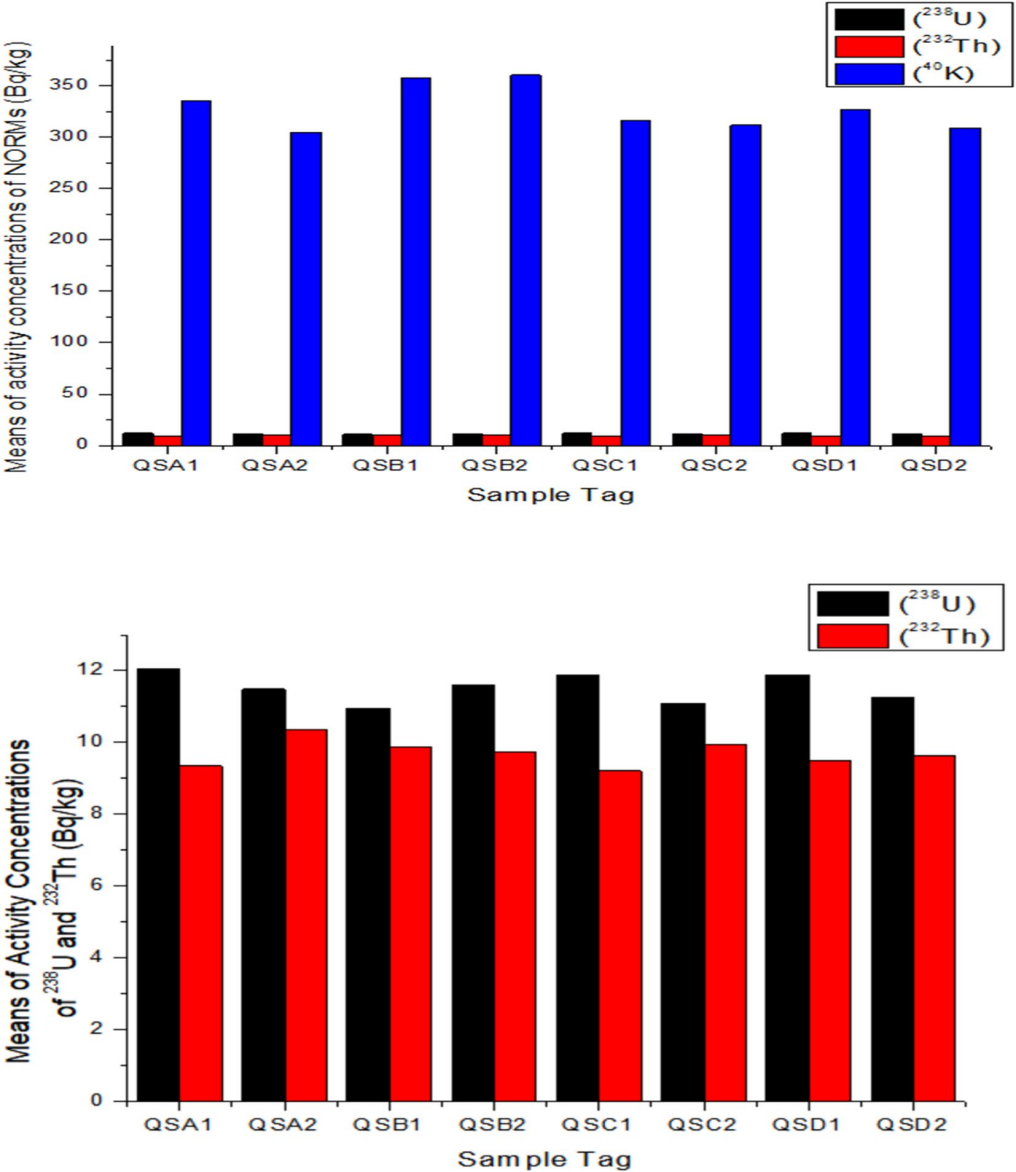
(**a**) Bar graph depicting the activity levels of the NORMs in the studied area. (**b**) Bar graph depicting the activity levels of ^238^U and ^232^Th in the studied area.

The ranges and mean values of the determined radioactivity concentrations of ^40^K, ^232^Th, and ^238^U are reported in columns 3–6 of Table 3, respectively. Sample tag QSA1 represents five samples obtained from quarry site A (QSA), while QSA2 represents neighboring soil samples obtained 2–5 km from QSA, and so on. The results of the radioactivity concentration indicate that the quarry products were enriched with various radioactive minerals. The range of the measured activity concentrations of the three natural radionuclides from the quarry products and the surrounding soil samples were found to be comparable (as featured graphically in Figs. 2a & b). For example, the measured activity level of ^238^U in QSA1 ranges from 10.61 ± 3.4 Bq/kg to 13.19 ± 3.0 Bq/kg, with an average value of 12.05 ± 1.1 Bq/kg, while the range for QSA2 is from 10.13 ± 4.2 Bq/kg to 12.80 ± 2.3 Bq/kg, with a mean value of 11.48 ± 1.1 Bq/kg. This comparison shows that quarry activities neither enhance NORMs concentration in the studied quarry sites nor alter the NORMs status of the surrounding soil.

**Table 3:** Range and mean of radioactivity levels, radium equivalent activity, absorbed dose rate, annual effective dose (indoor, outdoor, and total), hazard indices, activity indices, and the gonadal dose equivalent of the sampled quarry products.

No of samples	Sample Tag		Radioactivity levels $(Bq/kg)$					Effective dose $(mSv\:{y}^{-1})$			Hazard indices		Activity indices		
			^238^U	^232^Th	^40^K	$\:{Ra}_{eq}(Bq/kg)$	$\:{D}_{R}(nGy/h)$	*A*_*doutdoor*_	*A*_*dindoor*_	*A*_*dtotal*_	*H*_*in*_	*H*_*ex*_	*I*_*γ*_	*I*	$\:{D}_{GE}\:\:\:\left(\mu\:Sv\:{y}^{-1}\right)$
5	QSA1	Min	10.61± 3.4	8.42*±* 3.1	300.09± 1.1	49.35	45.79	0.06	0.22	0.28	0.16	0.13	0.19	0.07	172.70
		Max	13.19± 3.0	10.93*±* 5.4	378.30± 10.0	53.63	50.76	0.06	0.25	0.31	0.18	0.14	0.21	0.08	191.58
		Mean (± SD)	**12.05± 1.1**	**9.35± 1.1**	**334.82± 41.9**	**51.20**	**48.15**	**0.06**	**0.24**	**0.30**	**0.17**	**0.14**	**0.20**	**0.07**	**181.43**
5	QSA2	Min	10.13± 4.2	9.74± 2.0	265.62± 22.1	47.32	44.18	0.05	0.22	0.27	0.16	0.13	0.18	0.07	166.51
		Max	12.80± 2.3	11.18± 3.2	346.31± 2.4	52.83	49.68	0.06	0.24	0.30	0.18	0.14	0.20	0.08	187.28
		Mean (± SD)	**11.48± 1.1**	**10.35± 1.0**	**304.82± 28.9**	**49.76**	**46.34**	**0.06**	**0.23**	**0.28**	**0.17**	**0.13**	**0.19**	**0.07**	**174.47**
5	QSB1	Min	10.17± 1.1	9.10± 1.1	322.13± 10.0	47.99	45.14	0.06	0.22	0.28	0.16	0.13	0.19	0.07	170.61
		Max	12.21± 2.2	11.23± 3.2	398.10± 20.1	57.06	53.72	0.07	0.26	0.33	0.18	0.15	0.22	0.09	203.93
		Mean (± SD)	**10.95± 1.0**	**9.87± 1.0**	**357.54± 33.1**	**52.60**	**49.53**	**0.06**	**0.24**	**0.30**	**0.17**	**0.14**	**0.21**	**0.08**	**187.36**
5	QSB2	Min	10.13± 4.2	9.17± 2.3	268.10± 25.1	43.92	40.88	0.05	0.20	0.25	0.15	0.12	0.17	0.06	153.90
		Max	12.80± 2.3	10.62± 2.1	425.21± 12.1	60.73	57.47	0.07	0.28	0.35	0.20	0.16	0.24	0.09	217.46
		Mean (± SD)	**11.60± 1.0**	**9.73± 1.0**	**360.25± 59.8**	**53.25**	**50.19**	**0.06**	**0.25**	**0.31**	**0.18**	**0.14**	**0.21**	**0.08**	**189.62**
5	QSC1	Min	10.61± 2.1	8.42*±* 3.6	310.09± 1.2	48.15	45.19	0.06	0.22	0.28	0.16	0.13	0.19	0.07	170.36
		Max	13.19± 3.0	10.33± 5.4	328.30± 3.1	50.28	47.15	0.06	0.23	0.29	0.17	0.14	0.19	0.07	176.92
		Mean (± SD)	**11.87± 1.0**	**9.21± 1.0**	**316.21± 7.4**	**49.38**	**46.34**	**0.06**	**0.23**	**0.28**	**0.17**	**0.13**	**0.19**	**0.07**	**174.45**
5	QSC2	Min	10.13± 2.2	9.01± 2.1	271.12± 22.1	48.15	44.60	0.05	0.22	0.27	0.16	0.13	0.18	0.07	166.99
		Max	12.80± 2.3	11.18± 3.3	331.12± 21.3	51.85	48.22	0.06	0.24	0.30	0.17	0.14	0.20	0.08	181.89
		Mean (± SD)	**11.09± 1.0**	**9.94± 1.0**	**311.70± 24.1**	**49.30**	**46.06**	**0.06**	**0.23**	**0.28**	**0.16**	**0.13**	**0.19**	**0.07**	**173.66**
5	QSD1	Min	10.61± 3.4	9.03± 3.2	302.12± 1.0	48.93	45.96	0.06	0.23	0.28	0.16	0.13	0.19	0.07	173.35
		Max	13.19± 3.0	10.12*±* 3.1	362.13± 10.1	53.15	50.27	0.06	0.25	0.31	0.18	0.14	0.21	0.08	189.62
		Mean (± SD)	**11.88± 1.0**	**9.51± 1.0**	**326.50± 22.1**	**50.61**	**47.50**	**0.06**	**0.23**	**0.29**	**0.17**	**0.14**	**0.20**	**0.07**	**178.95**
5	QSD2	Min	10.10± 1.2	9.11± 3.2	285.62± 2.1	47.87	44.91	0.06	0.22	0.28	0.16	0.13	0.19	0.07	168.65
		Max	12.00± 2.3	10.10± 1.0	321.12± 22.0	50.28	47.06	0.06	0.23	0.29	0.17	0.14	0.19	0.07	177.11
		Mean (± SD)	**11.24± 1.0**	**9.62± 0.5**	**308.74± 13.7**	**48.77**	**45.62**	**0.06**	**0.22**	**0.28**	**0.16**	**0.13**	**0.19**	**0.07**	**171.88**

The mean activity level of ^238^U was the lowest at SQB1, with 10.95 ± 1.0 Bq/kg, and peaked at SQA1, with 12.05 ± 1.1 Bq/kg. The minimum mean activity level of ^232^Th was recorded at SQC1, with 9.21 ± 1.0 Bq/kg, while the maximum was at SQA2, with 10.35 ± 1.0 Bq/kg. The average activity levels of ^40^K had the lowest value at SQA2, with 304.82 ± 28.9 Bq/kg, and the highest value at SQB2, with 360.25 ± 59.8 Bq/kg.

^40^K is observed to have the highest activity concentrations in the sampled quarries, although it remains below the global average of 400 Bq/kg, as suggested by the United Nations Scientific Committee on the Effects of Atomic Radiation^16^. High activity levels of ^40^K also suggest a high content of alkaline feldspar, as potassium concentrations have been verifiably linked with K-feldspar samples^45,46^. The radioactivity levels of ^232^Th and ^238^U were also established to be lower than the world averages of 30 and 35 Bq/kg, respectively^16^.

### Radium equivalent level and absorbed dose rate

The evaluated ranges and mean values of radium equivalent levels and the absorbed dose rate in the studied area are featured in columns 7 and 8 of Table 3, respectively. The mean values of radium equivalent levels peak at QSB2 (53.25 Bq/kg) and are lowest at QSD2 (48.77 Bq/kg), all below the suggested recommended limit of 370 Bq/kg by at least 85%^11,47^. The range of the mean values of the absorbed dose rate is from 45.62 nGy/h to 50.19 nGy/h. This indicates that the mean gamma doses in all the assays are substantially lower than the global average of 84 nGy/h by at least 40%^12,33^.

The results of the absorbed dose rate and radium equivalent level establish that quarry products from the studied site are radiologically safe for use in constructing structures or edifices intended for various human activities—residential, commercial, industrial, religious, recreational, educational, resort and hospitality, institutional, transportation, agricultural, and historical or cultural buildings. It also shows that the studied sites and their environments do not pose any radiation threat to workers on the sites, people frequenting the areas or nearby communities.

**Fig. 3:**
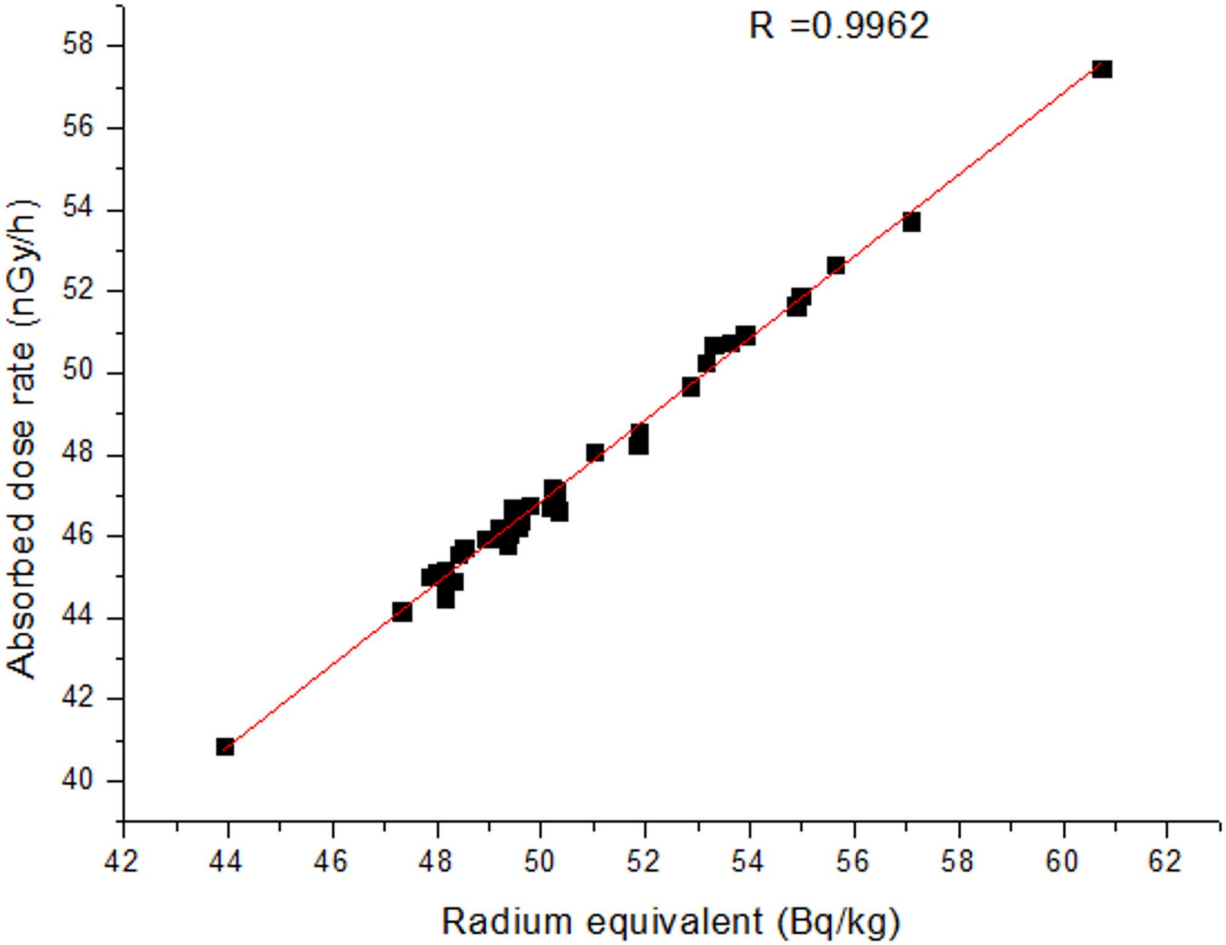
Coefficient of Correlation between the absorbed dose rate and radium equivalent level for the studied assays.

The connection between the radium equivalent level and the absorbed dose rate is shown in Fig. 3, with a correlation coefficient of *R* = 0.9962, indicating that the absorbed dose rate is significantly influenced by the gamma radiation emitted from NORMs.

### Effective dose rate

**Fig. 4:**
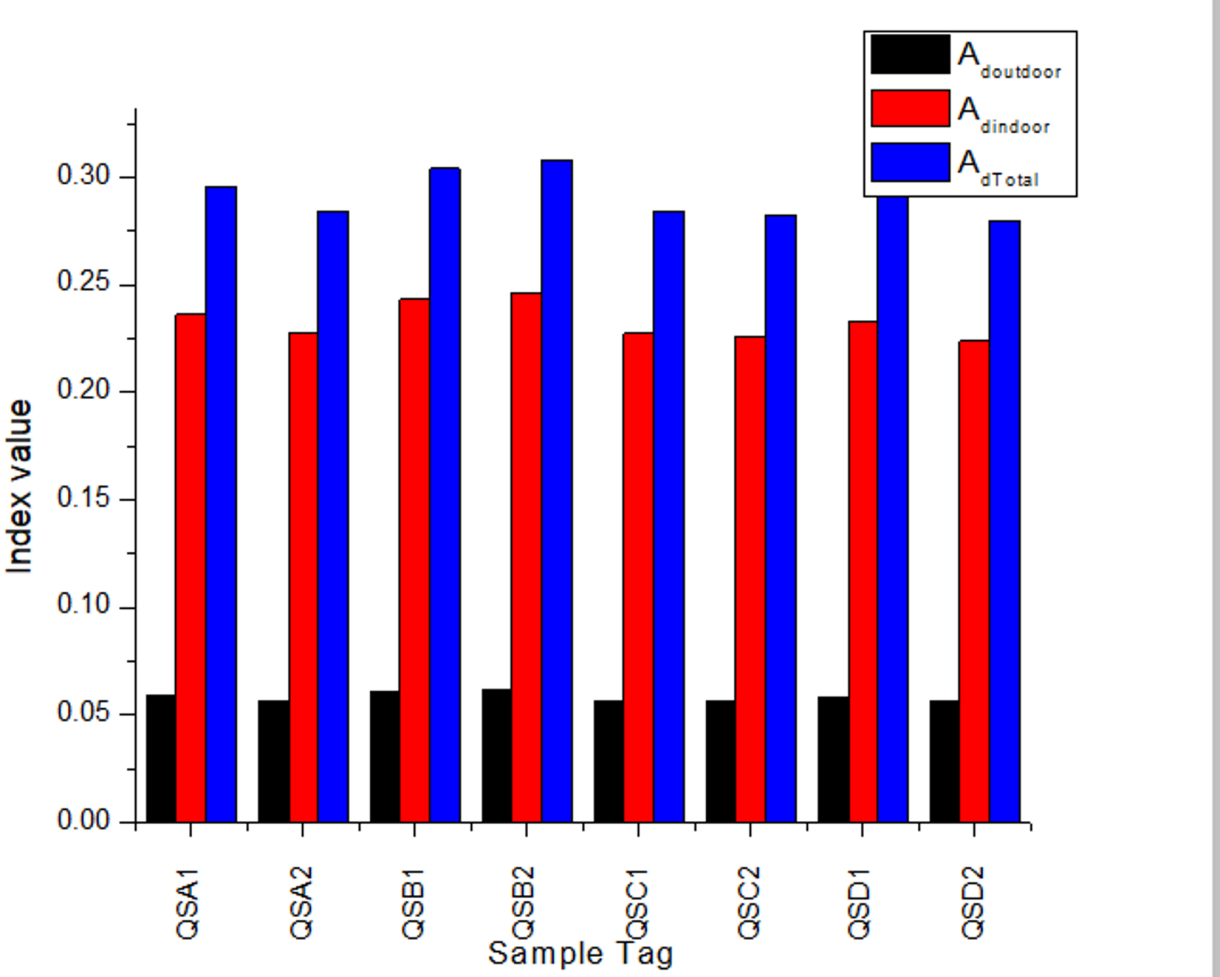
Graph depicting average effective dose.

Results of the evaluated outdoor, indoor, and total vulnerability to gamma rays from the studied samples are featured in columns 9–11 of Table 3. The outdoor effective dose rate has a maximum value of 0.07 mSv/y, which is equivalent to the worldwide average, at QSB1 and QSB2, and the minimum value of 0.05 mSv/y at QSA2, QSB2, and QSC2, with an average of 0.06 mSv/y. The calculated mean of the indoor effective dose rate ranges from 0.22 to 0.25 mSv/y, all below the global mean value of 0.41 mSv/y by at least 46.3%. The mean total effective dose rate ranges from 0.28 to 0.31 mSv/y, values below the global average of 0.49 mSv/y by a minimum of 42.8%^16^. Effective dose results are presented in the line graph in Fig. 4. These calculated results of the outdoor, indoor, and total effective dose rates suggest a negligible radiation threat from the environment from which the samples were sourced.

### Hazard and activity indices

The summarized results of the internal and external hazard indices are reported in columns 12 and 13 of Table 3. The average values of the internal hazard index range from 0.16 to 0.18, which are at least 82% below the recommended safety limit of 1^23^. The evaluated mean values of the external hazard index are 0.13 and 0.14, which are far smaller than the global acceptable safety limit. The evaluated radiation hazard indices indicate a minimal radiation risk from the quarry products at the studied sites.

The gamma radioactivity level index (I_γ_) and the activity utilization index (I) are presented in summarized form in columns 14 and 15 of Table 3. The gamma radioactivity level index (I_γ_) shows values at least 81% below the worldwide average value of 1, while the activity utilization index (I) values are at least 96.5% below the global mean value of 2^34,39^. This indicates negligible radiation hazards from exposure to quarry products from the studied sites and their surrounding soils.

### Gonadal dose equivalent $({\varvec{D}}_{\varvec{G}\varvec{E}})$

The summarized evaluated result of the gonadal dose equivalent per annum (D_GE_) is featured in column 16 of Table 3. The calculated mean values of the gonadal dose equivalent per annum range from 171.88 µSv/y for QSD2 to 189.62 µSv/y for QSB2, which are at least 36.79% below the recommended average of 300 µSv/y^16,40^. This result affirms that the studied quarry sites, quarry products, and their surroundings are radiologically safe.

### Excess lifetime cancer risk (ELCR)

Abridge results of evaluated excess lifetime cancer risk (ELCR) is presented in Table 4. Columns 4, 5, and 6 feature the outdoor, indoor, and total excess lifetime cancer risk, respectively. The minimum outdoor excess lifetime cancer risk is observed to be $\:0.14\times\:{10}^{-3}$ while the maximum is $\:0.19\times\:{10}^{-3}$, all lower than the worldwide average of $\:0.29\times\:{10}^{-3}$^35^. The indoor excess lifetime cancer risk range is from $\:0.55\:\times\:{10}^{-3}$ to $\:0.78\times\:{10}^{-3}$, values with a maximum of $\:67.24\:\%$ global averages. The evaluated total excess lifetime cancer risk is observed to be lower than the recommended limit by at least $\:33.10\:\%$. All these affirm an insignificant contribution to excess lifetime cancer risk from exposure to natural radiation emanating from the studied quarry sites and products and their surroundings.

**Table 4:** Range and mean of evaluated excess lifetime Cancer risk (ELCR) from the sampled quarry products.

Number of samples	Sample tag		$\:{ELCR}_{outdoor}\:(\times\:{10}^{-3})$	$\:{ELCR}_{indoor}\:(\times\:{10}^{-3})$	$\:{ELCR}_{Total}\:(\times\:{10}^{-3})$
5	QSA1	Min	0.15	0.62	0.77
		Max	0.17	0.69	0.86
		Mean	0.16	0.65	0.81
5	QSA2	Min	0.15	0.60	0.75
		Max	0.17	0.67	0.84
		Mean	0.16	0.63	0.78
5	QSB1	Min	0.15	0.61	0.76
		Max	0.18	0.73	0.91
		Mean	0.17	0.67	0.84
5	QSB2	Min	0.14	0.55	0.69
		Max	0.19	0.78	0.97
		Mean	0.17	0.68	0.85
5	QSC1	Min	0.15	0.61	0.76
		Max	0.16	0.64	0.80
		Mean	0.16	0.63	0.78
5	QSC2	Min	0.15	0.60	0.75
		Max	0.16	0.65	0.81
		Mean	0.16	0.62	0.78
5	QSD1	Min	0.16	0.62	0.78
		Max	0.17	0.68	0.85
		Mean	0.16	0.64	0.80
5	QSD2	Min	0.15	0.61	0.76
		Max	0.16	0.64	0.79
		Mean	0.15	0.62	0.77

### Statistical analysis

Multivariate datasets often contain hidden information, which can be summarized with essential insights extracted using appropriate statistical tools. These tools use multivariate classification procedures to identify common features among groups of variables. Large heterogeneous groups are divided into smaller homogeneous groups, where members share similar characteristics and are distinct from other groups^46^.

In this study, statistical analysis for data management and interpretation was performed using SPSS Statistics 26. This was done to reduce data dimensionality and identify key elements contributing to significant variance within the set of measured elemental concentrations^11^. Correlation statistics were applied to the assessed concentrations of radioactive elements and the evaluated associated risks to determine the connectivity and strength of associations between pairs of variables. The results are presented in the correlation matrix in Table 5.

**Table 5:** Correlation matrix among the measured and the evaluated quantities.

	^238^U	^232^Th	^40^K	Ra_eq_	D_*R*_	A_doutdoor_	A_dindoor_	A_dTotal_	H_in_	H_ex_	I_γ_	I	D_GE_
^238^U	1.0000												
^232^Th	-0.1480	1.0000											
^40^K	0.0837	-0.0025	1.0000										
Ra_eq_	0.3333	0.2927	0.9053	1.0000									
D_R_	0.3220	0.2169	0.9346	0.9964	1.0000								
A_doutdoor_	0.3220	0.2169	0.9346	0.9964	1.0000	1.0000							
A_dindoor_	0.3220	0.2169	0.9346	0.9964	1.0000	1.0000	1.0000						
A_dTotal_	0.3220	0.2169	0.9346	0.9964	1.0000	1.0000	1.0000	1.0000					
H_in_	0.5641	0.2161	0.8157	0.9665	0.9603	0.9603	0.9603	0.9603	1.0000				
H_ex_	0.3336	0.2926	0.9053	1.0000	0.9964	0.9964	0.9964	0.9964	0.9666	1.0000			
I_γ_	0.2830	0.2475	0.9367	0.9967	0.9989	0.9989	0.9989	0.9989	0.9499	0.9966	1.0000		
I	0.0785	0.3129	0.9479	0.9653	0.9680	0.9680	0.9680	0.9680	0.8668	0.9652	0.9781	1.0000	
D_GE_	0.2863	0.2177	0.9447	0.9945	0.9993	0.9993	0.9993	0.9993	0.9489	0.9945	0.9995	0.9761	1.0000

In Table 5, ^238^U and ^232^Th are negatively and weakly correlated, with *R* = -0.1480. Similarly, ^232^Th and ^40^K are weakly correlated, with *R* = -0.0025, indicating independent sources. The radioactivity level of ^40^K is strongly correlated with all evaluated radiation hazard indices (*R* > 0.8), showing a strong dependency of all indices on the activity levels of ^40^K. ^232^Th and ^238^U are weakly correlated with all the calculated radiological hazard indices, with 0.0025 ≤ *R* ≤ 0.3336, implying that the activity levels of these elements influence the assessed radiation hazards. All the appraised radiological risk indices are positively and strongly correlated (*R* > 0.9) with each other, suggesting they can be attributed to the same source.

### Hierarchical cluster analysis (HCA)

Hierarchical cluster analysis (HCA) is a technique of data classification that employs multivariate techniques to identify authentic data clusters. In clustering, similar variables are grouped, and observations with the highest number of similarities are combined first. This procedure is replicated until all observations are classified. A dendrogram is then constructed based on the similarity at which observations are merged^48–50^.

**Fig. 5:**
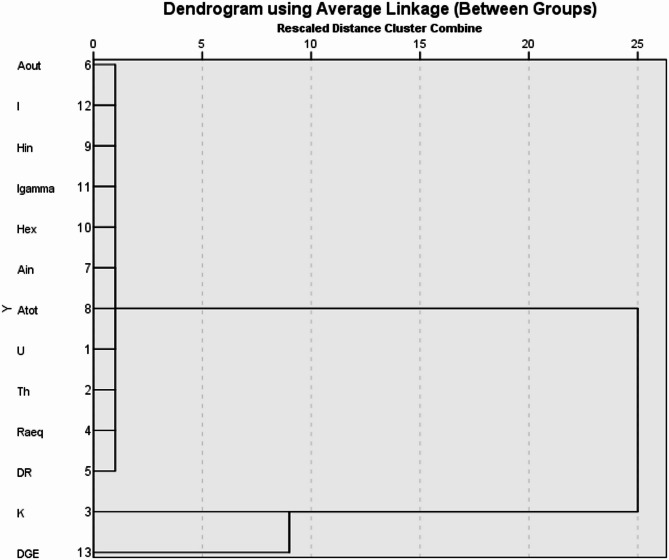
Dendrogram showing the clustering of measured activity concentration and the evaluated radiation risk parameters.

The degree of similarity between measured activity concentrations and the evaluated radiation risk parameters was determined in this work and is presented as a dendrogram in Fig. 5. The derived dendrogram for all 13 measured and calculated quantities is classified into two statistically significant clusters. Cluster I consists of ^238^U, ^232^Th, and other radiation risk parameter distributions, such as A_out_, I, H_in_, I_gamma_, H_ex_, A_in_, A_tot_, Ra_eq_, and D_R_. This implies that the distribution of these radiation risk parameters in the studied quarry sites and their surroundings largely depends on the radioactivity levels of ^232^Th and ^238^U. Cluster II consists of ^40^K and G_DE_, indicating a significant dependence of the gonadal dose equivalent per annum on the concentration of ^40^K.

## Conclusion

An assessment of quarry products from four quarry sites and their surrounding soils in Osun State, Southwestern Nigeria, was conducted using the gamma-ray spectroscopy method. This was done to determine the radioactivity levels of ^40^K, ^232^Th, and ^238^U in the evaluated samples and assess the associated radiological risks these may pose to the exposed population. The surrounding soils of the studied quarry sites were also examined to assess potential radiation threats and the possible influence of quarry activities on the radiation levels of the quarry products and the environment.

The results confirm the presence of these natural radionuclides in all the samples, though at concentrations below the global averages of 35, 30, and 400 Bq/kg for ^238^U, ^232^Th, and ^40^K, respectively. The activity levels of ^238^U ranged from 10.95 ± 1.0 to 12.05 ± 1.0 Bq/kg, ^232^Th from 9.21 ± 1.1 to 10.35 ± 1.0 Bq/kg, and ^40^K from 304.82 ± 28.82 to 360.25 ± 59.8 Bq/kg. This indicates that the examined quarry products and the surrounding soils are radiologically safe for use in all forms of construction.

The measured activity concentrations of ^40^K, ^232^Th, and ^238^U from the examined quarry sites were also found to be comparable to those of the peripheral soils, suggesting that the radiation levels of the quarry products, sites, and surroundings due to the assessed radionuclides were not affected by quarry activities. The average values obtained for the associated radiological risks—effective dose rates (outdoor: 0.06 mSv/y, indoor: 0.23 mSv/y, and total: 0.29 mSv/y), absorbed dose rate (47.47 nGy/h), radium equivalent level (50.68 Bq/kg), gamma radioactivity level index (0.2), internal (0.17) and external (0.13) hazard indices, activity utilization index (0.07), and gonadal dose equivalent per annum (178.98 µSv/y)—were all lower than globally acceptable hazard thresholds. This indicates that no significant radiation threat arises from the examined quarry products and their peripheral soils. Therefore, these materials are deemed radiologically safe for use in the construction of private and commercial buildings, as well as for other construction purposes. The results of the excess lifetime cancer risks also corroborate this fact.

Statistical analysis results associate the variations in the calculated radiological risk indices in the studied quarry products and surrounding soils with the concentrations of the three natural radionuclides.

## Data Availability

The datasets generated and analyzed during this study are available from the corresponding author upon reasonable request.
